# Applications of Space Technologies to Global Health: Scoping Review

**DOI:** 10.2196/jmir.9458

**Published:** 2018-06-27

**Authors:** Damien Dietrich, Ralitza Dekova, Stephan Davy, Guillaume Fahrni, Antoine Geissbühler

**Affiliations:** ^1^ Hopitaux Universitaires de Genève eHealth and Telemedicine Division Geneva Switzerland

**Keywords:** satellite imagery, satellite communications, public health, remote sensing technology, global positioning system, geographic information systems, telemedicine, spaceflight, space medicine, global health

## Abstract

**Background:**

Space technology has an impact on many domains of activity on earth, including in the field of global health. With the recent adoption of the United Nations’ Sustainable Development Goals that highlight the need for strengthening partnerships in different domains, it is useful to better characterize the relationship between space technology and global health.

**Objective:**

The aim of this study was to identify the applications of space technologies to global health, the key stakeholders in the field, as well as gaps and challenges.

**Methods:**

We used a scoping review methodology, including a literature review and the involvement of stakeholders, via a brief self-administered, open-response questionnaire. A distinct search on several search engines was conducted for each of the four key technological domains that were previously identified by the UN Office for Outer Space Affairs’ Expert Group on Space and Global Health (Domain A: remote sensing; Domain B: global navigation satellite systems; Domain C: satellite communication; and Domain D: human space flight). Themes in which space technologies are of benefit to global health were extracted. Key stakeholders, as well as gaps, challenges, and perspectives were identified.

**Results:**

A total of 222 sources were included for Domain A, 82 sources for Domain B, 144 sources for Domain C, and 31 sources for Domain D. A total of 3 questionnaires out of 16 sent were answered. Global navigation satellite systems and geographic information systems are used for the study and forecasting of communicable and noncommunicable diseases; satellite communication and global navigation satellite systems for disaster response; satellite communication for telemedicine and tele-education; and global navigation satellite systems for autonomy improvement, access to health care, as well as for safe and efficient transportation. Various health research and technologies developed for inhabited space flights have been adapted for terrestrial use.

**Conclusions:**

Although numerous examples of space technology applications to global health exist, improved awareness, training, and collaboration of the research community is needed.

## Introduction

### Background

The space-earth frontier is no longer afforded to a narrow niche of individuals. Compared with over 50 years ago when the first humans reached outer space, and satellite function only concerned a small number of scientists, today many programs and research projects in multiple fields exist that make use of outer space technologies. The field of global health too—interdisciplinary by definition—has innovated over the years and has made strides in the advancement of health aims using space technologies. Examples include using remote sensing technology to detect environmental changes that have a significant effect on local population health, satellite communication for medical endeavors and management of natural disasters, advancing medical knowledge through space medicine programs, and tapping into the benefits of localization through global navigation satellite systems (GNSSs). The UNISPACE+50 conference, taking place in 2018, marks the 50th anniversary of the start of the United Nations (UN) conferences that engaged states to cooperate in their outer space engagements. After half a century of cooperation and innovation, it is an appropriate time to take stock of where the global health field has ventured into its use of space technologies.

### Objectives

The Expert Group on Space and Global Health of the UN Office for Outer Space Affairs (UNOOSA), in its 2016 work plan, mandated one of its members, Antoine Geissbühler, to produce a compilation of practices and initiatives [1] in the form of a scoping review, including both a literature review and stakeholders’ involvement, via a self-administered questionnaire to identify (1) The main stakeholders in the field, (2) The key applications of space technologies to global health, and (3) The gaps, challenges, and perspectives.

This work uses a scoping review methodology, including both a literature review and stakeholders’ involvement via a self-administered questionnaire. These are used to identify (1) The main stakeholders in the field, (2) The key applications of space technologies to global health, and (3) The gaps, challenges, and perspectives.

Key stakeholders of the fields are first briefly presented. Then, main themes in which space technologies are of benefit to global health are identified and illustrated in four technological domains. Finally, findings are summarized, and gaps, challenges, and perspectives are discussed.

## Methods

### Scoping Review

The general aim of a scoping review is to “map rapidly the key concepts underpinning a research area and the main sources and types of evidence available and can be undertaken as [a] stand-alone project in [its] own right, especially where an area is complex or has not been reviewed comprehensively before” [2]. As opposed to systematic reviews, scoping reviews can include a diversity of sources and, in particular, are not necessarily limited to scientific articles. This allows researchers to gain a better overview on a broad subject but prevents precisely answering a well-defined question.

Accordingly, the scoping review methodology matches our objectives and was chosen for this work [3,4]. The Expert Group on Space and Global Health identified four key technological domains that are applied or could be applied to global health [5,1]: *domain A:* remote sensing, *domain B:* GNSS, *domain C:* satellite communications, and *domain D:* human space flight. Our scoping work was conducted using these domains as a framework. A distinct literature search was conducted for each of the four key technological domains on PubMed, with eventual further insights gathered from RERO, the Western Switzerland online network for libraries, and Google Scholar. Additionally, stakeholders’ insights were collected through an emailed, self-administered questionnaire.

### Literature Review

#### Search Strategy

Searches were conducted per technological domain. PubMed was the main search engine used. Complementary searches were performed on RERO and Google Scholar. Resources retrieved by these search engines were included only if they brought insights that were not identified in the original search. The keywords used for each domain are listed in Table 1. For each domain, the basic search structure was “domain-associated technology” AND “health.” Medical Subject Headings (MeSH) terms were not systematically used as some did not properly refer to the technology we were searching for. For Domain C, the search term “eHealth” was used in addition to “health” as it is a MeSH entry term for “telemedicine.” The year-parameter of the search was unbounded to access published material that could date back to the start of outer space technology and its application to global health activities. The “Similar Articles” feature of PubMed, as well as the list of references of included articles were used to identify additional resources. Finally, key stakeholders’ websites were assessed for ongoing projects (listed in Multimedia Appendix 1).

#### Material Inclusion

Presentations, books, websites, and articles identified by the searches were included if they satisfied all of the following criteria:

1. Reporting research, or an applied program related to health
2. Use of space technology based on one of the four domains (remote sensing, GNSS, satellite communication, and inhabited space flight)
3. Only for RERO and Google Scholar: global health application not already described in a resource identified through the PubMed search

In each domain, duplicates found across the various search engines were excluded. Included resources were entered in Endnote (Clarivate Analytics) by domain and exported on spreadsheets (one for each domain). Importantly, resources written in languages other than English but whose abstracts were translated to English were included in the review. However, for these, full texts were not read.

#### Analysis and Reporting

On the spreadsheets described above, global health applications were identified for each resource. Then, main themes of global health applications per technological domain were identified. The numbers of resources per theme were counted in an attempt to weight the different themes (Tables 2-5) for a particular technological domain. Articles dealing with more than one theme were allocated according to the dominant theme. If this was not possible, they were classified as “miscellaneous.” The different themes were then described by domain in the main text and illustrated by the citation of relevant articles.

**Table 1:** Search keywords used in this study.

Domain	Keywords used
Remote sensing	Remote sensing Health
Global navigation satellite systems	Satellite Global Positioning System (GPS) Global Navigation Satellite Systems (GNSS) Geographic Information Systems (GIS) Health
Satellite communication	Satellite communication Satellite Telemedicine Global Health Health eHealth
Inhabited space flight	Human spaceflight Manned spaceflight

#### Stakeholder Involvement

To gather additional insights, a brief semistructured, self-administered questionnaire (Multimedia Appendix 2) was created and sent by email to 16 stakeholders identified after the initial literature searches. Two reminders were eventually sent to nonresponders. The questionnaire was created following the same structure and logic as our overall work. Four open-ended questions were used, asking participants about:

1. Key applications of space technologies to global health for each domain and eventual other domains
2. Gaps, challenges, and opportunities
3. Key events related to the topic
4. Other important remarks they may have

Comments on the current state of the space-technology- global-health interface are included at the end of the Results section, whereas gaps and potential solutions are presented in the Discussion section.

## Results

### Data Collected

After the whole literature review process, 222 articles were included for domain A, 82 articles for domain B, 144 articles for domain C, and 31 articles for domain D. In total, 473 articles were included (6 of those were included in 2 domains). Most of the included resources were peer-reviewed scientific articles (96%, 213/222 for domain A; 99%, 81/82 for domain B; 84%, 121/144 for domain C; and 100%, 31/31 for domain D), and other types of sources included mainly book sections and Web pages. The mean publication year and the minimal and maximal publication years were 2010 (1985; 2017) for domain A, 2010 (1996; 2016) for domain B, 2004 (1986; 2016) for domain C, and 1999 (1981; 2011) for domain D. Of note, in accordance with the scoping methodology used for this work and described in the Methods section, we used different combinations of keywords; included resources via the “Similar Articles” feature of PubMed and the list of references of included articles and navigation on stakeholders’ websites.

Regarding questionnaires, 3 out of 16 sent were answered and included for the analysis.

### Presentation of the Stakeholders

Using insights from the literature review and the questionnaires, we performed a nonexhaustive listing of stakeholders implicated in the space and global health fields.

**Figure 1:**
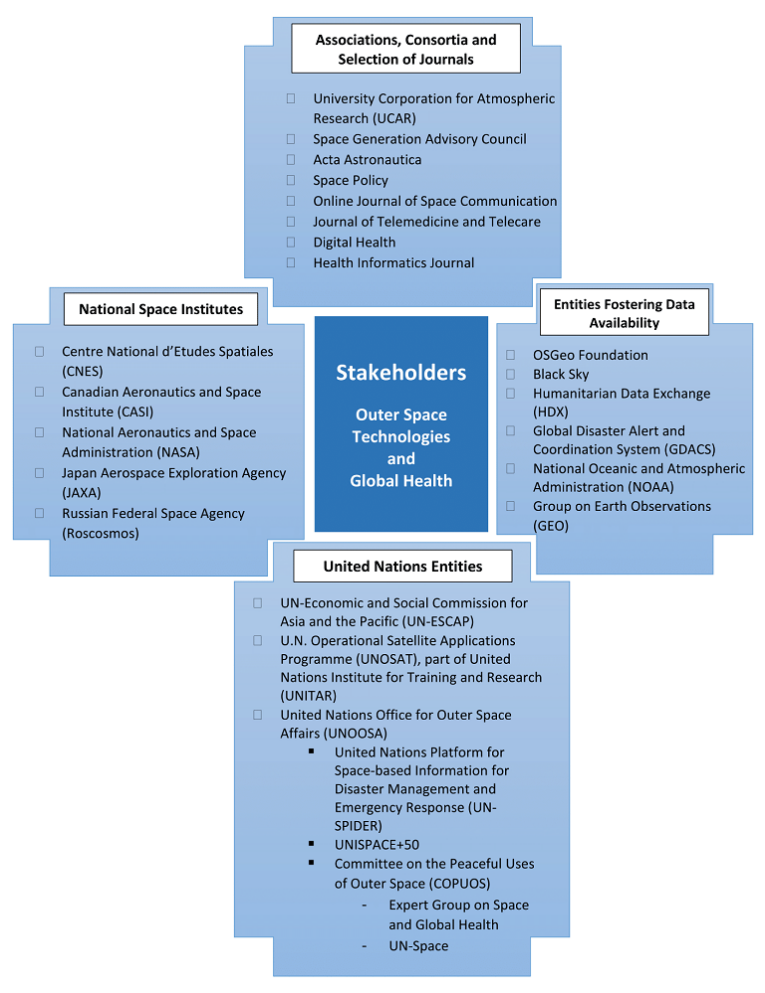
Nonexhaustive collection of stakeholders and journals in the intersection of space technology and global health.

We categorized stakeholders per their nature: National Space Institutes; UN entities and specialized agencies; entities fostering data availability, usage, analysis and exchange; and journals, other consortia, and associations. These stakeholders are depicted in Figure 1.

National Space Institutes are usually public institutes that are responsible for applying their countries' spatial programs. Their missions are space exploration, education, research, and development that can sometimes be translated into commercial applications, or eventually for terrestrial use. Nonexhaustively, we identified the US’s National Aeronautics and Space Administration (NASA), the Russian Federal Space Agency, the Japan Aerospace Exploration Agency, the French Centre National d’Etudes Spatiales, and the Canadian Aeronautics and Space Institute as being engaged at the space and global health interface.

The UN comprises several entities that deal with space and global health. The UN platform for Space-based Information for Disaster Management and Emergency Response (UN-SPIDER) and the UN Operational Satellite Applications Program (UNOSAT) aims at providing all countries and international organizations with space-based information useful for disaster risk management and emergency response. This is also one of the goals of the UN Economic and Social Commission for Asia and the Pacific. UN-Space is an interagency coordinating body aiming at fostering collaboration and synchronization between the various agencies implicated in space and global health. The Committee on the Peaceful Use of Outer Space (COPUOS) was set up by the general assembly in 1959 to govern the exploration and use of space for the benefit of all humanity: for peace, security, and development. The Expert Group on Space and Global Health that guided this review is part of COPUOS and has a focused scope on global health applications of space technologies. Of note, UNOOSA is a governing office that comprises UN-SPIDER, UN-Space, and COPUOS. It is also in charge of organizing the UNISPACE+50 conference that will mark the 50th anniversary of the first UN conference on the peaceful uses of outer space that engaged states to cooperate in their outer space uses. Applications of space technologies to global health is also an important interest of the World Health Organization (WHO), a specialized UN agency.

In addition, we identified entities aiming at fostering satellite data availability, analysis, visualization, interoperability, and exchange. As an example, the Group on Earth Observations (GEO) is a partnership of governments and organizations whose one activity among others is to build the Global Earth Information System of Systems. This platform offers access via a Web-based interface to earth-observation data coming from multiple sources, including satellites. It acts as a connector between different data sources and thus, increases data availability for researchers, public health professionals, and international organizations. The Global Disaster Alert and Coordinating System is a cooperative framework under the UN umbrella that connects to various services and platforms (the majority of which are listed in this section) to create a comprehensive solution that aims to create early alerts in the case of a disaster, to assess the impact of the disaster, to coordinate the response, and to provide disaster maps and satellite images. Black Sky is a service of Spaceflights Industries (a private company) that provides access to satellite imagery in addition to other sources of data (eg, radio communication and social media). It also offers spatial analysis based on those datasets and several algorithms. Humanitarian Data Exchange is an open platform for data sharing in the humanitarian context. The OSGeo foundation is a foundation that supports the creation and usage of an open source geospatial software. Finally, the National Oceanic and Atmospheric Administration provides environmental data, some of which are acquired via satellites. It is to be noted that most of the national space institutes listed previously are data providers too.

Some stakeholders are consortiums or associations active in the field of space and global health. We included the University Corporation for Atmospheric Research that regroups North American colleges and universities focused on research and training in the atmospheric and related Earth system sciences. The Space Generation Advisory Council is a nongovernmental organization that promotes the access of students and young professionals to UN agencies and National Space Institutes.

Finally, we included as part of Figure 1 a nonexhaustive list of journals that are implicated in the field of space and global health.

### Domain A: Remote Sensing

#### Definition

Remote sensing refers to data collection at distance, usually from a satellite or an aircraft, as opposed to on-site sensing.

#### How It Works

A sensor, carried by a satellite or an aircraft, detects electromagnetic radiation coming from Earth and its characteristics. The electromagnetic radiation may be the reflection of an external source of energy (usually the sun) or of a source of energy carried by the satellite or aircraft itself. The terms passive or active remote sensing are used, respectively [6].

The detected signal is then processed through algorithms of various complexities to derive the parameters of interest. Example of parameters that can be derived via remote sensing include land temperature, altitude, humidity, rainfall, cloud coverage, air pollutants, livestock density, vegetation indices, sea temperature, sea salinity, sea nutrient concentration, sea algae concentration, sea bacteria concentration, urbanization, population density, and bare soil coverage. This list is nonexhaustive.

#### Insights From the Literature Review

Overall, remote sensing was useful for global health in three major ways:

- Identifying associations between diseases (or disease vectors) and remotely sensed parameters
- On the basis of these associations, model development and forecasting of the spatio-temporal evolution of diseases, thus allowing rational public health strategies
- Direct monitoring of certain microorganisms

Two major themes and two secondary themes were identified and are presented in Table 2.

Main themes of global health applications in the remote sensing domain were identified, and the total number of resources per theme were counted as described in the Methods section.

Remote sensing was most used to identify determinants of infectious diseases and to develop models to predict their evolution (Theme A-1). For example, Midekisa et al [99] quantified the degree of association between malaria cases and remotely sensed environmental parameters such as rainfall, vegetation indices, and temperature. On this basis, they developed and tested a model able to predict malaria evolution and thus, guide public health decisions. Applications of spatial technologies for malaria transmission modeling and control were reviewed in 2015 by Gebreslasie [48]. In addition to malaria [8-10,17,24,26,31,33-35,38-42,46,48,49,55,56,76,87, 90-93,99,100,102,103,105,111-115,118,119,122, 129-132,141] and schistosomiasis [15,37,53,54,63,95,124,126, 127,142-145,153,154,156,158], dengue fever [7,12,13,16,23, 44,89,94,98,101,117,140], cholera [43,71,72,74,75,80,88], and cyanobacterias [28,81,82,123,137,138,148,150,155] were the most studied.

**Table 2:** Main themes of global health application for the remote sensing domain.

Theme category		Theme	Articles identified
**Main themes-A**			
	A-1	Infectious diseases	153 [7-159]
	A-2	Air pollutants and noncommunicable diseases (NCDs)	37 [160-196]
**Secondary themes-B**			
	B-1	Other environmental pollutants and NCDs	8 [197-204]
	B-2	Other environmental parameters and NCDs	12 [205-216]
	B-3	Miscellaneous	12 [217-224,6,225-227]

Other studied diseases or pathogens included meningitis [225]; brucellosis [70]; C. *imicola* [67]; avian pathogens [25,134,136,50]; V. *vulnifucus* [52]; V. *parahaemoliticus* [52]; *Fasciola hepatica* [36]; hand, foot, and mouth disease [20]; Helminth infections (not limited to schistosomiasis) [120,85,21,22]; Lyme disease [108,45,79,110]; Guinea worm [30]; Nipah virus [133]; onchocerciasis [68]; opistorchiasis [146]; rotavirus [69]; typhoid fever [32]; Rift Valley fever [139,125,84]; Murray Valley encephalitis virus [121]; West Nile fever virus [96,159]; and hanta virus [149,152].

In an important number of studies, disease vectors (and not disease cases) were the outcomes predicted based on sensed environmental parameters. These vectors included Anopheles [10,55,103,141] (transmitting malaria) and Aedes [16,44,101,117] (transmitting dengue) mosquitos, as well as ticks [45,64,77,79,110,147] (transmitting Lyme’s disease among other tick-borne diseases).

Of note, remote sensing techniques allow to directly derive the concentrations of some bacteria. Cyanobacteria produce various toxins that have been linked to the occurrence of amyotrophic lateral sclerosis and nonalcoholic liver disease [155,138]. They also have distinct fluorescent properties that can be exploited in active remote sensing to monitor their concentration [28,81,82,123,137,138,148,150,155].

The second main theme (A-2) was the use of remote sensing to monitor air pollutants and eventually link them to noncommunicable diseases (NCDs) such as respiratory diseases (asthma [161,188,191] and others [185,162,208]), coronary artery disease [165], premature birth [195], and low birth weight [181]. Particulate matter (PM2.5 and PM10) [188,181,161,195, 196,175,194,193,164,189,174,170,165,163,173,171,172,160,178,183], O_3_[218], NO_2_[180,225,218], pollens [209], asbestos [202], volcanic ash [184], and wildfire smoke [168,190,176] are among the air pollutants that can be effectively detected by remote sensing. Temperature and humidity are usually included as additional parameters when monitoring air pollutants as they may affect both respiratory diseases and air pollutants behavior. If many articles successfully describe the use of remote sensing for the monitoring of air quality, only a few establish a direct link between air pollutants and health outcomes [188,162,181,191,161,195,165,185,208]. Moreover, results may be controversial, such as for asthma, where one study found a correlation between childhood asthma hospital admission that disappears after multivariate analysis [161], another one finds no correlation between air pollution and asthma prevalence [188], and a last one finds a correlation between PM2.5 concentration and salbutamol (treatment used in asthma and chronic obstructive pulmonary disease) use [191].

The remaining articles identified for remote sensing dealt with monitoring environmental pollutants (B-1) or parameters (B-2) and their links with NCDs. For example, studies investigated the link between urban greenness and birth outcomes [212] or cardiovascular diseases [206]. Others investigated the link between drought and respiratory illnesses [208] or between heat and elderly health [215,211] or childhood diarrhea [205]. Additional parameters or pollutants that can be sensed by remote sensing include artificial lights [207], soil contaminants (heavy metals [203], nitrates, nitrogens [197]), water quality [128,97,14], and arsenic [198].

### Domain B: Global Navigation Satellite System

#### Definition

GNSS is the generic term for satellite navigation systems that provide autonomous geo-spatial positioning with global coverage [228]. GNSSs are satellite ensembles that allow any user on or near the Earth to determine their position with a precision from some meters to some centimeters.

The term global positioning system (GPS) is specific to the US’ GNSS, the NAVSTAR GPS. The Global Orbiting Navigation Satellite System (GLONASS) is the Russian Federation’s GNSS. As of 2013, these two are the only fully operational GNSSs.

Other GNSSs in various stages of development and deployment include:

- Galileo, the European Union’s positioning system
- IRNSS, India’s next generation regional system
- QZSS, the Japanese regional system
- China’s BeiDou (COMPASS) GNSS

**Table 3:** Main themes in the global navigation satellite systems (GNSS) domain.

Theme category		Theme	Articles identified
**Main themes-C**			
	C-1	Noncommunicable diseases	22 [230-251]
	C-2	Communicable diseases	18 [252,253,25,254,255,38,48,256-258,95,259-265]
	C-3	Innovative methods for research	26 [266-291]
**Secondary themes-D**			
	D-1	Autonomy improvement	6 [292-297]
	D-2	Transportation	5 [298-302]
	D-3	Health care access	4 [303-306]
	D-4	Accurate timekeeping	1 [307]

#### How It Works

Each system (GPS, GLONASS, Galileo, COMPASS, etc) consists of a constellation of satellites that send a continuous signal toward the Earth. Individuals wanting to use GNSS to determine their position must have an antenna that receives the signals coming from the satellites and a receiver that translates these signals. The antenna position will be deduced from the measurements of the time delay between the emission time (satellite) and the reception time (receiver) for at least four signals coming from different satellites [229]. Most importantly, the atomic clocks onboard the satellites are all synchronized so that the signals coming from the different satellites of the same constellation share the same reference time scale.

Although a GNSS is the space technology that is highlighted in this review, often mentioned in global health applications is the use of a geographic information system (GIS). A GNSS allows a user to determine the location of an object or individual, whereas a GIS is the system for storing, combining, and displaying data (partly coming from GNSS) on a map. It allows users to easily visualize spatial data, analyze them, and interpret trends or patterns.

#### Insights From the Literature Review

Seven themes were identified after the literature review and are shown in Table 3.

Main themes of global health applications in the GNSS domain were identified, and the total number of resources per theme were counted as described in the Methods section.

GNSS was used in epidemiological studies, often in combination with GIS and remote sensing. NCDs were the focus of many studies, whether directly as a measured outcome, or because of their risk factors being studied [239,236,232,245]. Physical activity (PA) was a very popular research area [243,237,247,233,250], most notably in children and adolescents [230,242,240,248,234,244,231]. Edwards and authors [234] assessed adolescents’ use of public parks with regards to the features of the parks. The parks were characterized using GIS and a desktop auditing tool that uses remote sensing techniques, whereas the adolescents were surveyed to assess their activities. In two other studies in the United States and Switzerland [244,231], participants wore GPS receivers and accelerometers, enabling researchers to assess and compare the intensity and location of the PAs. Links between different locations (home, playground, sidewalk, and more) and the intensity of PA were identified. In addition to PA, the built and natural environment were studied for their associations with NCDs. Researched environmental determinants of health ranged from air pollution [291,251] and water quality monitoring [246,241] to the complex ways in which climate change impacts global health [259]. For this purpose, researchers used GNSS and satellite imagery in a variety of ways. Interestingly, happiness was also studied as a health outcome. MacKerron and Mourato (2013) [238] used GPS to locate individuals at various, spontaneous moments while they answered questions about their subjective well-being. They found that participants were substantially happier in natural rather than urban environments. The variety of ways in which GIS can be used in environmental epidemiological studies was reviewed by Nuckols et al (2004) [282], who concluded that GIS and GPS are useful tools in providing precise locations of subjects and studying proximity and level of exposure to environmental contaminants.

GNSSs have been used often in the field of communicable diseases too, including person-to-person transmissible varieties [253,255], vector-borne diseases [21,48,38,252,258,260, 257,264,256,95,263], and zoonoses [262,260,254,261,25]. In our search, the most studied communicable disease was malaria. Predicting vector breeding sites [21,38,257], malaria incidence, and adherence to medication [263] using GNSS, often in combination with GIS and remote sensing, were some practical applications. Additionally, distance to health facility was also used for malaria risk mapping [265]. Studies of zoonotic communicable diseases were limited to avian pathogens in this domain. Newman et al [260] marked two hosts of H5N1, a highly pathogenic avian influenza, with GPS transmitters and found links between flu outbreaks in humans and the hosts’ travel patterns.

The use of GNSS as a new tool for epidemiological research was discussed in a variety of articles [282,275,270,274, 280,279,281,287,288,271,269,284,268]. GNSS use was reported to construct random sampling frames for surveys, mapping households, or determining population estimates [267,278, 272,277,273,266,285,289,276]. The potential future impacts of GPS devices on medicine is discussed in Pager’s article, *Impacts for medicine of global monitoring* [283].

Geolocation of individuals has been used is in the assistance of mentally or physically impaired individuals to improve their autonomy [294,297]. Alisky [293] presents hypothetical scenarios whereby GPS devices can be of assistance. For instance, in the case of an individual with partial complex seizure disorder, the individual can wear a GPS-enabled watch that will notify a health management center in the case of a seizure. Gallay et al [292] give a review of GPS technologies that have already been available to aid visually impaired individuals to navigate their surroundings. They discuss several limitations, eg, that the GPS receiver does not work well unless satellite coverage is satisfactory, and this is affected by climatic conditions as well as the user’s location. GPS can also be of assistance for persons suffering from dementia. This could be achieved through orientation and safety cues, daily reminders of activities, protection against wandering, and direct links to medical assistance in case of incapacitation. Potential benefits are decreased stress and workload for formal and informal caregivers, decreased institutionalization, and thus, lower costs.

Geolocation is also helpful in promoting health care access in different settings [306,303]. In Bolivia, Perry et al [305] used GPS techniques and satellite imagery of the remote, impoverished, and mountainous region of Andean Bolivia to create a GIS that enabled them to assess the physical accessibility of several populations to health care services and auxiliary nurses [305]. Their findings demonstrate how medical geography can be used for better informed health care policy and planning decisions. Tassetto et al [286] tested a novel method to locate victims of disaster by using their existing portable devices (such as simple mobile phones or laptops) and the existing cellular network. Their proposed technology is mediated by a satellite system and requires little action by victims. Although this new system has been tested in experimental settings, it is yet to be used in real-life scenarios. In northern Nigeria, polio vaccination teams were tracked with handheld GPS devices, and their movements were overlaid on catchment area maps [304]. This method allowed the identification of low vaccine coverage areas and was identified as a tool to improve microplanning of global health projects.

The use of GNSS to improve transportation for improved public health appears as one area in which there is a huge potential for growth, for instance by preventing road accidents. Guo et al [301], working under the current constraints of suboptimal space-time reference for vehicles, conducted research with the aim of locating vehicles with high precision, down to the lane in which the vehicle is moving. This has immense safety implications which, in addition to a safety notification system, can provide information on high-risk vehicles (eg, trucks carrying chemicals) or high-priority-of-way vehicles (eg, school busses) and can also track illegal or dangerous vehicle movements [301]. Other transportation-related GPS studies have focused on speeding [300], commute routes, and daily mobility [299,298], as well as emergency patient transportation [302].

Finally, in a category of its own, accurate timekeeping using GPS was a proposal brought forth by Aljewari et al, especially in settings where time is of utmost importance, such as in hospitals [307].

### Domain C: Satellite Communication

#### Definition

Satellite communication is the ability of information to travel from one area to another via a communication satellite that is in orbit around the Earth. It is often performed with mobile satellite phones and is distinct from cellular phones that use earth-based towers that form a cellular network. “Wide area coverage, reliable data delivery, and robustness and broadcast or multicast are the unique features of satellite systems” [308].

#### How It Works

Satellite communication has two main components: the ground segment, which consists of fixed or mobile transmission, reception, and ancillary equipment, and the space segment, which primarily is the satellite itself. A typical satellite link involves the transmission (uplinking) of a signal from an Earth station to a satellite. The satellite then receives and amplifies the signal and retransmits it back to Earth (downlinking). Satellite receivers on the ground include direct-to-home satellite equipment, mobile reception equipment in aircraft, satellite telephones, and handheld devices [309].

#### Insights From the Literature Review

This domain was largely centered on telemedicine, often combined with tele-education. Health-on-the-go is defined below with several examples from the literature, and there are a handful of demonstrations of how satellite communication can be of importance in disaster situations. Main themes are presented in Table 4.

Main themes of global health applications in the satellite communication domain were identified, and the total number of resources per theme were counted as described in the Methods section.

Telemedicine is the application of communication technologies to the field of health in instances where medical expertise or resources are not available on site for different reasons. These reasons, nonexhaustively, include the geographical distance; physical barriers (mountains, space, desert, etc) and insufficient time or resources to transfer a patient. Often, the patient may be in the physical presence of a health care provider (HCP), but telemedicine could mean connecting the two parties to a third party at a distance, such as a medical specialist or a general practitioner (GP) if the HCP is a nonphysician. Telemedicine is possible via satellite and cellular network. This review is limited to telemedicine by means of satellite communications. More in-depth assessment of the definition and breadth of telemedicine can be found in several review and discussion references [390,386,337,393,441,383,352,353], some theoretical articles linking satellite communication with health [441,415, 446,448,372,444,451,453,449,445,442,454,447,364,450], as well as country reports [330,387,322,378,343,355,359,325, 321,399].

**Table 4:** Main themes in the satellite communication domain.

Theme category		Theme	Articles identified
**Main themes-E**			
	E-1	Telemedicine	90 [310-399]
	E-2	Tele-education	14 [400-413]
	E-3	Health-on-the-go	14 [414-427]
	E-4	Disaster prevention, early warning, and management	13 [428-440]
**Secondary themes-F**			
	F-1	Miscellaneous	13 [441-453]

A first example is in Thailand, where the country’s first communication satellite, THAICOM, was launched in 1993. HCPs in rural areas were connected with specialists in urban areas, and consultations became possible, with two main components: videoconferencing and exchange of medical images. Thailand’s telemedicine network is housed in its Ministry of Public Health, with all hospitals that are in the telemedicine network also having a direct communication link with the government base. The Thai example illustrates a common model of telemedicine and teleconsultation: access to expert opinion by GPs, nurses, or paramedics via videoconferencing or textual exchange [373,368, 315,318,316,397,351,314]. These are often accompanied by still images from radiography [332,361,311] or dermoscopy [356], but innovative advances have made possible the transfer of 3D images [381] and live ultrasound feed [331,374,317,326]. Use of telemedicine methods has been reported in various medical fields including dermatology [345], pediatrics [327], and surgery. Telesurgery [376] has been trialed on internal mammary artery dissection in pigs with robotic technology to determine feasibility and bandwidth requirements. The authors concluded that telesurgery via satellite communication is feasible and also identified the limit of satellite bandwidth below which it cannot be performed (3 Mb/s).

Telemedicine using satellite communication may also be useful for a country’s defense system. By equipping more than 300 US Navy ships with telemedicine capabilities, researchers estimated that 17% of medical evacuations could be avoided, representing US $4400 savings per single medical evacuation [384]. Similarly, German defense units have access to a telemedicine workstation, accompanied by a medical officer present on-board the ship or at the unit [371]. This station has the possibility of being equipped with various medical devices (X-ray film digitizer, dermatoscope, otoscope) and can also contain other imaging methods (eg, videocamera and ultrasound). The authors propose cooperation not only between civilian and military health service providers but also military-military cooperation between the medical services of allied armed forces.

As the field of telemedicine is both broadly defined and applied, as well as having fluid borders with tele-education and health-on-the-go, further sources were found in this search that do not fall under the broader categories discussed above [310,312,313,319,320,323,324,328,329,333-335,338,339,341,342,344, 347-350,354,357,358,360,362,363,365,369,370,375,377,379,380,382, 385,388,389,412,391,392,394,452,395,398].

Medical tele-education, the practice of providing new or continuing medical education via distance learning, often uses the same networks and infrastructures as telemedicine does. It is especially useful for HCPs who are located far from teaching facilities [321,400,401,403-411]. The Réseau en Afrique Francophone pour la Télémédecine network is one such example of successful implementation of tele-education; a model that has expanded into multiple countries and continents [402]. Health educators, usually located in teaching universities of larger cities of the region, teach courses to HCPs in peripheral areas in real time. Two-way communication enables students to ask questions and collaborate with the lecturer. Exchanges in the same country or between neighboring countries are promoted as much as possible to build capacity and collaboration. Another application of tele-education is implemented in Japan, where 39 universities and institutes were connected by satellite for a joint radiology conference [413]. Participants engaged in discussions around various images, and the results of a survey to radiologists after the conference showed that while the technology used may not be good for diagnosing purposes, it is useful for discussion and educational purposes.

The third broadly studied area of satellite communication and global health is what we refer to as health-on-the-go. In this theme, which can be considered as subcategory of telemedicine, mobile medical units can provide treatment and can transmit health information (text, health parameters, images, laboratory exams) using satellite communication [426,427,421,419,422,414,418]. This gives the ability to provide health care services to individuals over a large area that may be deprived of traditional communication systems. The TraumaStation is one such device, a portable and lightweight suitcase that carries ultrasound, electrocardiogram, blood pressure, and oxygen meter apparatus [425]. The TraumaStation allows for telecommunication with instant messaging and real-time video stream through satellite and a variety of other gateways. Alternatively, the HOPEmobile provides biometric measurement (body mass index, cholesterol, glycosylated hemoglobin, and retinal screening) from a mobile unit [416]. The study reported a return on investment of US $15 for every US $1 spent and a significant reduction in overall cholesterol at the second screening of a patient. Finally, Guo and colleagues (2015) [417] describe a portable, robust, and low-power device that performs all essential functions of enzyme-linked immunosorbent assay and can thus diagnose diseases in remote, mobile contexts. The results can then be sent via cell phone short message service (SMS) messaging or in email format via satellite. The authors describe how patient confidentiality is taken into account through the usage of this device. Another area of health-on-the-go is emergency patient transportation. The transmission of the patient’s medical history, vital signs, and laboratory exams (for instance electrocardiogram) during the transport can allow a remotely based medical expert to guide the management of the patient. Nakajima et al [424] explain that 3G mobile networks tend to be sensitive to congestion in urban areas and that the satellite provides a good solution to counter this. One technical innovation in this area includes the Emergency Medical Video Multiplexing Transport System. This divides a patient’s live video stream from a medical vehicle into four pieces, and these translate to high-quality videos that can be viewed by emergency doctors in a remote location [423].

Satellite communication is also valuable in emergency situations arising from natural disasters, man-made disasters (eg, terrorism and war), highly contagious diseases, or large-scale epidemics [431,440,436,437,433,432,430,429,435,439,434]. Satellites for Epidemiology (SAFE) is a system for early health warnings in a postdisaster period. It is a system that combines satellite, radio, wireless networks, and GIS to promptly identify and respond to a disease outbreak. SAFE’s added value is reported to be its integration into already-existing national, regional, and international preparedness plans [428]. Existing cellular and telephone networks almost always become overloaded or disabled following disasters, so satellite communication methods are superior in these instances. For this reason, East Carolina University tested the time it would take to set up a fault-tolerant communications infrastructure from scratch; one component of several being the satellite connection. They concluded that the time it took to mount the network by technically trained personnel made it a feasible and valuable contribution to disaster response operations. Potential drawbacks of this are that technical experts of the system may need to be made a part of the team of emergency responders and that in case of loss of electrical power, alternative methods need to be used [438].

### Domain D: Human Space Flight

#### Definition

We looked for evidence on how inhabited space flight-associated technologies and procedures may promote global health.

#### Literature Review

Two main themes and one secondary theme were identified and are represented in Table 5. Main themes of global health applications in the inhabited space flight domain were identified, and the total number of resources per theme were counted as described in the Methods section.

Telemedicine seems to be one of the dominant theme at the crossings of inhabited space flights and global health. Indeed, providing health care for an astronaut needing medical assistance onboard a space station, or an individual living far from medical expertise in a desolated rural area, may pose similar problems. In both cases, one must deal with the restriction of not being able to quickly transfer the patient and limited medical resources and expertise in the patient’s vicinity [366,462,463,465,467,466,468]. Telemedicine thus provides a possible solution in both cases. Interestingly, challenges for successful implementation are similar in space and on earth. They include dealing with low bandwidth connection, maintaining stable electrical power, assuring data storage, developing intelligent software, and training users.

Going further in the similarities between space and earth telemedicine, tele-ultrasound has been extensively designed and tested in space [460,458,459,457,455,456,461,480] but is also used on earth [484]. In addition, tele-surgery has been developed and practiced on earth [376] and is foreseen to be a requirement to medical support in extraplanetary human outposts [469]. Challenges for this particular implementation notably include the latency between the command and the robot movement, induced by the long distance [469]. Taken together, telemedicine in space and telemedicine on earth are facing similar yet complementary challenges that are potential synergies for researches in the development, implementation, and testing phases.

Among included articles, technology transfer of space technologies to earth appears to be an important topic [470,429,471-479,481]. An example is the successful reprogramming of neural networks initially trained to identify craters or incoming missiles in space toward the detection of cancer-associated breast microcalcifications on mammograms [478,479,474]. The potential use, on Earth, of miniature or implantable biometric sensors developed by the NASA sensors 2000! program (S2K!) is another example of technology transfer [472].

The space scientific community is actively conducting research on how to provide adequate life support for long extraterrestrial missions or on extraplanetary outposts. In addition to new technology transfers, outputs from this research should lead to development of new medical procedures that may be applicable on earth [483].

This review focuses on how inhabited space flight-associated technologies and procedures may promote global health. It is important to note that, in addition to global health, the space research community has also been very active in various domains of life sciences. These domains include microgravity physiology, microgravity microbiology, microgravity surgery, radiation medicine, and the study of the psychological effects because of space travel and isolation.

### Insights From the Questionnaires

Respondents’ insights were collated and are reported below.

#### Domain A: Remote Sensing

Stakeholders believe that incorporating environmental exposure data into clinical practice will improve the quality of care. Indeed, diagnostic accuracy may be improved via integration of remotely sensed parameters into decision support tools. For example, knowing that the allergens concentration was high over the last days will increase the probability of asthma when a patient consults for breathlessness.

**Table 5:** Main themes in the inhabited space flight domain.

Theme category			Theme	Articles Identified
**Main themes-G**				
	**G-1**		Telemedicine	16
		G1-1	Tele-ultrasound	7 [455-461]
		G1-2	General telemedicine	8 [462-466,366,467,468]
		G1-3	Tele-surgery	1 [469]
	G-2		Technology transfer	11 [470,429,471-479]
**Secondary themes-H**				
	H-1		Application of medical procedures	4 [480-483]

#### Domain B: Global Navigation Satellite Systems

No insights were provided by respondents.

#### Domain C: Satellite Communications

Stakeholders provided more examples about situations in which telemedicine is used through satellite networks. These situations include people onboard a plane, on a boat, working on an off-shore platform, or on construction sites. Satellite communication may also be necessary to provide telemedicine in remote areas of developed countries. Examples include communications between French overseas territories and the mainland, or locally between islands. In all these cases, satellite communications can be used to compensate for the unavailability of the cellular network.

#### Domain D: Human Space Flight

In this domain, in particular, stakeholder questionnaires provided insightful additions. Physical inactivity is a major determinant of NCDs such as cardiovascular diseases and osteoporosis. It is thus of particular interest to global health. Despite this fact, studies on the physiological effects of physical inactivity are lacking. In space, astronauts are exposed to microgravity, and accordingly, the space research and development area has been very active in studying the physiological effects of microgravity, notably by using ground-based bed rest analog. As microgravity partly mimics physical inactivity and aging, space-associated study results may help us to understand the deleterious physiological effects behind those processes. Future joint research programs should thus be encouraged.

As another example of space technology transfer, bone quality measurement tools were initially developed by the space industry. A NASA review of spin-offs of space research can be found on their website.

Long-term missions will require the development of “integrated countermeasures” to prevent the adverse effects of the space environment, including radiation. These countermeasures may find applications on earth, such as in radiation medicine.

Another challenge is to be able to personalize space medicine, which is a major trend in nowadays medicine. Moreover, when thinking about long flight duration, space health systems will need to achieve some level of autonomy, which imply the development of decision algorithms and consistent procedures that may be of benefit to global health, especially in isolated settings.

Finally, another big challenge of human space exploration is to develop a closed-loop environmental system technology to maintain, at low cost, an environment suitable for human life. These technologies include monitoring and control of physical, chemical, and biological environments; waste recycling; and food production. Results from such research may contribute to the development of sustainable and green solutions of benefit to global health.

## Discussion

### Principal Findings

Using a scoping review methodology, including a literature review and questionnaires to stakeholders, we identified, described, and illustrated key areas in which space technology is, or may be, of benefit to global health. Remote sensing of environmental parameters allows the prediction of communicable and NCD evolution, often in association with GIS and GNSS. GIS and GNSS are also used to bring new insights to epidemiological research, to improve access to health care, to develop autonomy assistance for the disabled, and to assist in disaster response. For this last task, space communications are also used, as well as in telemedicine and tele-education. Finally, some technologies and procedures developed by the space industry for inhabited-space flights are applied on earth. Overall, our results strengthen the vision that space technologies and global health are two synergistic fields, and they help us to identify perspectives and issues for the coming years that will be discussed in this section.

### Remote Sensing

Remote sensing brings new tools for monitoring diseases, investigating their association with multiple sensed parameters, and ultimately creating an intelligent alert system. The literature is particularly abundant on infectious diseases and air pollutants. One limit is that most studies do not link directly sensed parameters to health outcomes but rather to some disease determinants (disease vectors, air pollutants). This is an interesting first step as it gives insight to more than one disease. Yet, more studies investigating direct health outcomes are needed to allow the creation of relevant models that will guide public health decisions. Importantly, the limited presence of environmental monitoring systems in low-income countries is an obstacle. Moreover, achieving high spatial and temporal resolution either by hardware improvement or through the development of numerical models is an important challenge in remote sensing. Finally, the sustainability of the developed alert systems, as well as their reproducibility across different geographical areas, must be evaluated. In addition to adding value at the population level, remote sensing used in combination with GNSS holds great potential to assist caregivers in their routine decision making for individual patients. This could be done, eg, by assessing relevant environmental data for each patient.

### Global Navigation Satellite Systems

The last example illustrates the synergy between remote sensing, GIS, and GNSS applied to global health. Indeed, most of the epidemiological studies identified in this review and aiming at predicting disease evolution based on environmental parameters are using GIS and GNSS, in addition to remote sensing. GNSS and GIS are also used in innovative epidemiological methodologies for activity tracking (eg, movement or localization as an outcome or a determinant), randomization, or population estimation. Disaster response and autonomy improvement of disabled patients constitute two other fields in which these technologies are used. Requirements for the successful use of GNSSs are stable and easily accessible signals, as well as procedures preventing power failure. Combining space-, cyber-, and ground-data thus holds a great potential. The use of big data analytics and machine learning may lead to further applications that are not even suspected nowadays. Creating a platform warranting availability, interoperability, and quality of data issued from different sources is a requirement to go further in this direction.

### Satellite Communications

Satellite communications are mainly used when standard telecommunications using landlines and antenna are not available, such as in disaster situations. Through these networks, telemedicine and health tele-education are possible. Bringing medical expertise at distance is useful in various places such as in isolated rural areas, areas affected by natural disaster, but also elderly homes, isolated places in high-income countries (northern Canada, Alps), ambulances, and remote work places (off-shore platforms, boats, airplanes). In addition to information exchanges, telemedicine encompasses laboratory exams and medical procedures at distance and sometimes in real time. Examples include tele-echography, tele-electrocardiogram, tele-dermatoscopy, and tele-surgery.

### Human Space Flight

In parallel, research in outer space has been very active in developing telemedicine, including tele-echography and tele-surgery. In space and on earth, challenges for the development, implementation, and testing of telemedicine are similar and complementary. Strengthening existing collaborations in the field and creating new ones thus appear particularly relevant. In addition to telemedicine, we retrieved from the literature evidence of technology and medical procedure transfers from the space industry to the health sector. However, the number of articles retrieved was small and is probably not a true reflection of all ongoing synergies. This was confirmed by the questionnaire results that identified additional examples and themes such as the use of microgravity to study the physiology of physical inactivity, which is a major and frequent determinant of NCDs. Overall, it seems that encouraging collaborations between the space and health sectors is of particular interest for this domain (inhabited space flights). Moreover, reinforcement of the scientific publishing and public communication is needed to strengthen the scientific community awareness of the existing synergies.

### Value Added From Questionnaires

In all collected questionnaires, the potential of space applications to improve global health was reported to suffer from a lack of awareness among health workers and space researchers. Moreover, a deficit in space-associated skills and knowledge was also reported for health researchers. More interdisciplinary collaboration and an easier access for health researchers to space technologies was expressed. Finally, a gap in organizational level activities was identified. Accordingly, efforts are reported to be necessary to:

- Raise awareness on the potential global health applications of space technologies
- Train researchers interested in the field
- Promote interdisciplinary collaborations
- Improve the organizational-level governance

Results from the questionnaires suggested the reinforcement of public communication and the organization of dedicated conferences and training sessions as a first step toward a more comprehensive solution. Moreover, early involvement of end users and policy makers in the various projects has been suggested to improve their relevance.

### Implications of the Research

By providing a thorough review of the published literature on space and global health, as well as the identification of key stakeholders, this work presents a solid base for improving mutual understanding between the two domains. This should lead to more synergies among the various actors, including the development of formal interagency coordination mechanisms. Comprehensive strategies to address sustainable development goals must indeed leverage the complementary competencies from UN agencies such as the WHO, the UNOOSA, UNOSAT, as well as other organizations such as the GEO.

### Limitations

This review has several limitations. A scoping review is a methodology useful to gather as many insights as possible on a broad subject, such as this one, to achieve a better awareness of the question and its past and ongoing research, practices, and initiatives. We chose this methodology as it matches our objectives well. The searches that we ran gave us thousands of hits but only came from two search engines: PubMed and RERO Western Swiss database. Moreover, only 3 of the 16 questionnaires sent were answered despite two reminders. Accordingly, we can’t exclude that eventual supplementary themes were missed. In addition, the low response rate to the questionnaire may have introduced biases in the insights that were reported. Insights gathered from the questionnaires should thus be considered as expert opinions. Another limitation to our study is that we cannot draw definitive conclusions on precise subthemes and questions (eg, is remote sensing effective in predicting malaria outbreaks in Africa?). For this purpose, systematic reviews are needed. The different domains that guided the searches were suggested by the Expert Group on Space and Global Health. This group is mainly constituted by key stakeholders of various national space agencies and public health authorities. Accordingly, it is unlikely that an important domain was missed, but it constitutes a limitation to our study. The language barrier is another one. Indeed, the space literature in Russian or Japanese is abundant and not always available in an English translation, save for the abstract. Accordingly, key concepts may have been missed.

As the paper has technology at its core, one must note that articles used in the review date back to 1981. Space technology and access to it has improved significantly since then, but to remain aligned with the goal of the review, we reference all relevant articles. Nevertheless, as ease of use and access to space technology has improved in recent times, as well as an increased human presence in outer space, the themes will be largely shaped by more recent articles, simply as there are more of them.

## Appendix

Multimedia Appendix 1 List of stakeholder websites consulted for projects and meetings.

Multimedia Appendix 2 Questionnaire sent to stakeholders.

## Abbreviations

COPUOS: Committee on the Peaceful Uses of Outer Space
GEO: Group on Earth Observations
GIS: geographic information system
GLONASS: global navigation satellite systems
GNSS: global navigation satellite systems
GP: general practitioner
GPS: global positioning system
HCP: health care professional
MeSH: Medical Subject Headings
NASA: National Aeronautics and Space Administration
NCD: noncommunicable disease
PA: physical activity
PM: particulate matter
SAFE: Satellites for Epidemiology
UNOSAT: United Nations Operational Satellite Applications Program
UN-SPIDER: United Nations platform for Space-based Information for Disaster Management and Emergency Response
UNOOSA: United Nations Office for Outer Space Affairs
WHO: World Health Organization
