# Supplementary material for: Applications of Space Technologies to Global Health: Scoping Review
Source: J Med Internet Res. 2018 Jun 27;20(6):e230. doi: 10.2196/jmir.9458 (PMC6041558; doi:10.2196/jmir.9458)
Supplement: Multimedia Appendix 1 [file jmir_v20i6e230_app1.pdf]

APPENDIX 1

List of stakeholder websites consulted for projects and meetings

1. Black Sky : https://www.blacksky.com/news/ , archived at http://www.webcitation.org/6xswYsaHa
2. Canadian Aeronautics and Space Institute (CASI): http://www.casi.ca/ , unable to be archived
3. Centre National d'Etudes Spatiales (CNES) : https://cnes.fr/en , archived at http://www.webcitation.org/6xsvdVjtu
4. Expert Focus Group on Space & Global Health (XFG-SGH) : http://at6fui.weebly.com/at6fui-background.html , archived at http://www.webcitation.org/6xsxl0UoR
5. Global Disaster Alert and Coordination System (GDACS) : https://gdacs-smcs.unosat.org/site/about , archived at http://www.webcitation.org/6xswxvbzy
6. Group on Earth Observations (GEO) : https://www.earthobservations.org/index2.php , archived at http://www.webcitation.org/6xsx81XSS
7. Humanitarian Data Exchange (HDX) : https://data.humdata.org/dataset , archived at http://www.webcitation.org/6xswfhAgO
8. Japan Aerospace Exploration Agency (JAXA): http://global.jaxa.jp/area/ , archived at http://www.webcitation.org/6xswAYPTE
9. National Aeronautics and Space Administration (NASA): https://www.nasa.gov/ , archived at http://www.webcitation.org/6xsw5h7D6
10. National Oceanic and Atmospheric Administration (NOAA) : http://www.noaa.gov/ , archived at http://www.webcitation.org/6xsx0zh7D
11. OSGeo Foundation : https://www.osgeo.org/projects/ , archived at http://www.webcitation.org/6xswQOidm
12. Russian Federal Space Agency (Roscosmos) : http://en.roscosmos.ru/ , archived at http://www.webcitation.org/6xswKRVaH
13. Space Generation Advisory Council: https://spacegeneration.org/ , archived at http://www.webcitation.org/6xsvPEnqP
14. U.N. Operational Satellite Applications Programme (UNOSAT) : http://www.unitar.org/unosat/ , archived at http://www.webcitation.org/6xsxM62Py
15. UN-Economic and Social Commission for Asia & the Pacific (UN-ESCAP) : http://www.unescap.org/our-work/ict-disaster-risk-reduction/space-technologies-and-gis-applications-sustainable-development/about , archived at http://www.webcitation.org/6xsxGJGtd
16. United Nations Institute for Training and Research (UNITAR) : http://www.unitar.org/ , archived at http://www.webcitation.org/6xsxX5Vqo
17. United Nations Office for Outer Space Affairs (UNOOSA) : http://www.unoosa.org/oosa/en/ourwork/index.html , archived at http://www.webcitation.org/6xsxfgVT8
18. University Corporation for Atmospheric Research (UCAR): https://www2.ucar.edu/ , archived at http://www.webcitation.org/6xsuzhLwK
