# Supplementary material for: Applications of Space Technologies to Global Health: Scoping Review
Source: J Med Internet Res. 2018 Jun 27;20(6):e230. doi: 10.2196/jmir.9458 (PMC6041558; doi:10.2196/jmir.9458)
Supplement: Multimedia Appendix 2 [file jmir_v20i6e230_app2.pdf]

APPENDIX 2

Questionnaire sent to stakeholders

1. To your knowledge, how are space activities and technologies of benefit to global health?

2. Which gaps have you identified in this space-global health domain and how would you suggest to fill those gaps?

3. In your opinion, which are some notable regional and international meetings or conferences for the community of people working on the space-global health exchange as defined above?

4. Do you have any additional remarks or thoughts?
